# iDISCO+ for the Study of Neuroimmune Architecture of the Rat Auditory Brainstem

**DOI:** 10.3389/fnana.2019.00015

**Published:** 2019-02-13

**Authors:** Paola Perin, Fabian F. Voigt, Philipp Bethge, Fritjof Helmchen, Roberto Pizzala

**Affiliations:** ^1^Department of Brain and Behavioural Sciences, University of Pavia, Pavia, Italy; ^2^Brain Research Institute, University of Zurich, Zürich, Switzerland; ^3^Center for Neurosciences, University of Zurich and ETH Zurich, Zürich, Switzerland; ^4^Department of Molecular Medicine, University of Pavia, Pavia, Italy

**Keywords:** choroid plexus, cochlear nucleus, inner ear, microglia, macrophage, auditory system, 4th ventricle, iDISCO+

## Abstract

The lower stations of the auditory system display a complex anatomy. The inner ear labyrinth is composed of several interconnecting membranous structures encased in cavities of the temporal bone, and the cerebellopontine angle contains fragile structures such as meningeal folds, the choroid plexus (CP), and highly variable vascular formations. For this reason, most histological studies of the auditory system have either focused on the inner ear or the CNS by physically detaching the temporal bone from the brainstem. However, several studies of neuroimmune interactions have pinpointed the importance of structures such as meninges and CP; in the auditory system, an immune function has also been suggested for inner ear structures such as the endolymphatic duct (ED) and sac. All these structures are thin, fragile, and have complex 3D shapes. In order to study the immune cell populations located on these structures and their relevance to the inner ear and auditory brainstem in health and disease, we obtained a clarified-decalcified preparation of the rat hindbrain still attached to the intact temporal bone. This preparation may be immunolabeled using a clearing protocol (based on iDISCO+) to show location and functional state of immune cells. The observed macrophage distribution suggests the presence of CP-mediated communication pathways between the inner ear and the cochlear nuclei.

## Introduction

The role of inflammation is increasingly recognized in cochlear damage (Kalinec et al., 2017) and central auditory responses (Fuentes-Santamaría et al., 2017). Therefore, the immune system is being targeted to get novel insights in the onset, progression, and variability of auditory disorders. Both the ear (Fujioka et al., 2014) and brain (Louveau et al., 2017) have undergone a paradigm shift from the idea of an “immune-privileged” organ (with barriers) to that of a tightly regulated neuroimmune communication network with defined points of entry and exit (Louveau et al., 2017). However, the involvement of neuroimmune barriers, and in particular the choroid plexus (CP), which has been clearly established in the brain (Marques et al., 2017) has been so far largely neglected in the frame of the auditory system, mainly for lack of methods to study them *in situ*.

The auditory brainstem and periphery are anatomically very complex (the inner ear is appropriately called a “labyrinth”). Moreover, the anatomy of cerebellopontine angle blood vessels (Kim et al., 1990) and CP (Tubbs et al., 2016) displays high interindividual variability. *In situ* imaging techniques such as CT or MRI allow morphological study of the system, but not characterization of cell populations. On the other hand, removing the brain from the temporal bone rips apart brainstem meninges and often distorts the lateral part of CP upon paraflocculus dislodgement from the bony recess. Both these problems are solved by employing a cleared preparation of the intact auditory system which allows *in situ* labeling and imaging of cell populations without physical sectioning.

Several clearing protocols allowing to image through bone have been established in mouse (Susaki and Ueda, 2016), where the whole cleared body may be labeled and imaged. Clearing rat brains has been proven more difficult but possible (Stefaniuk et al., 2016). Here, we have adapted the iDISCO+ clearing method (Renier et al., 2016) in order to clarify hindbrain and temporal bone of adult rats, and we used this preparation to characterize the neuroimmune structures surrounding the rat auditory brainstem.

## Materials and Methods

Experiments were performed on inbred Wistar rats average age: [49 ± 17 days (mean ± S.D.; *n* = 6)]. Animals were housed with 12 h/12 h light/dark cycle, food and water provided *ad libitum*. Experiments were performed during the light phase. This study was carried out in accordance with the recommendations of Act 26/2014, Italian Ministry of Health. The protocol (number 155/2017-PR) was approved by the Italian Ministry of Health and University of Pavia Animal Welfare Office (OPBA). All efforts were made to minimize number of animals used and animal suffering.

### Sample Preparation

For all experiments except blood vessel tracing, animals were anesthetized with diethyl ether until complete areflexia and transcardially perfused with heparinized Krebs solution until complete blood clearance, followed by 4% PFA in PBS. For blood vessel tracing, animals were anesthetized and decapitated without transcardiac perfusion; the brain was rapidly dissected out (3–6 min from decapitation to fixation) and immersed in 4% PFA. All samples were postfixed overnight in 4% PFA and then cryoprotected in 30% sucrose solution until sinking.

Cryoprotected samples containing the intact temporal bone were decalcified with buffered 10% EDTA in PBS (daily changed) until softening of the squamous temporal bone (3–4 weeks). Decalcified samples were embedded in 5% gelatin, trimmed, and halved. After trimming, gelatin was mechanically removed from the sample and clearing was performed using the iDISCO+ protocol (Renier et al., 2016). Samples (shown in Supplementary Figure S1 before and after clearing) were immunolabeled using rabbit anti-Iba1 (WAKO, 1:200) for macrophages/microglia, sheep anti-transthyretin (TTR; Abcam, 1:250) for CP epithelium and goat anti-rat IgG (Thermo Fisher, 1:200) for blood vessel lumen (as in, Liebmann et al., 2016). Species-matched Alexa-conjugated donkey secondary antibodies (Life Technology) were used at 1:200.

### Lightsheet Imaging

**Figure 1:**
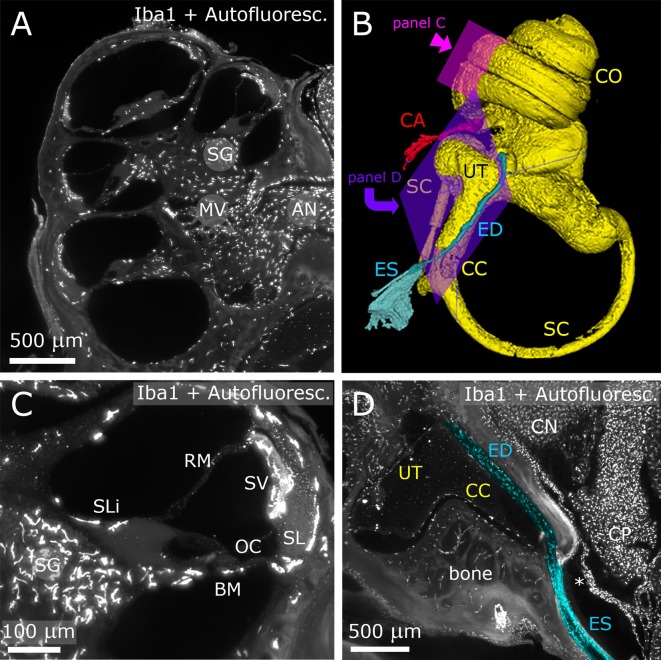
Inner ear. The fluorescence signal originates both from Iba-1 labeling and autofluorescence. Panels **(A,C,D)** show maximum projections from Z-stacks (**A**: 10 μm; **B**: 50 μm; **D**: 20 μm). **(A)** Representative section of the cochlea. **(B)** 3D reconstruction of the inner ear viewed from the medial side. **(C)** Higher magnification detail of a single cochlear turn. Same parameters as panel **(A)**. **(D)** Longitudinal section of ED and ES (pseudocolored blue) displaying macrophage populations. The ED follows CC and emerges at the inner surface of the bone as ES. A dural fold (asterisk) continuous with the periosteum separates ES from CP. AN, auditory nerve; BM, basilar membrane; CA, cochlear aqueduct; CC, crus commune; CN, cochlear nucleus; CO, cochlea; CP, choroid plexus; ED, endolymphatic duct; ES, endolymphatic sac; MV, modiolar vein; OC, organ of corti; RM, reissner membrane; SC, semicircular canal; SG, spiral ganglion; SL, spiral ligament; SLi, spiral limbus; SV, stria vascularis.

**Figure 2:**
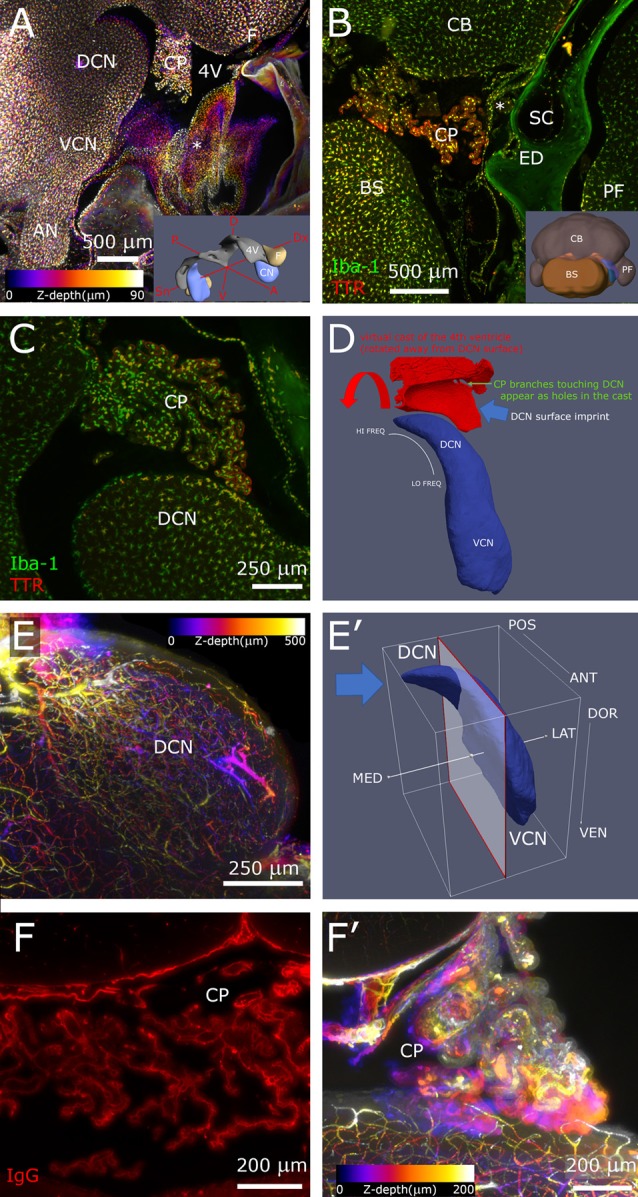
Hindbrain. **(A)** Sagittal z-projection of rat hindbrain showing the lateral exit of the 4th ventricle, partially covered by a meningeal fold (asterisk), pseudocolored with a Fire LUT (color corresponds to Z-depth relative to initial section). Inset: 3D model showing the relative positions of CN, 4th ventricle and flocculus. **(B)** Coronal optical section showing CP spatial relations at the DCN caudal end. Inset: 3D model (posterolateral view) showing CN position (blue) relative to ventricle (gray) and flocculus (beige). **(C)** Higher magnification sagittal optical section showing a CP branch associated to the DCN surface. **(D)** Segmented 4th ventricle volume (red), showing holes corresponding to CP-DCN contacts. CN model (blue; Muniak et al., 2013) is shown to explain the “virtual mold” shape features. **(E)** DCN vascular labeling. Z-stack of 500 optical sections (500 μm), pseudocolored as panel **(A)**. **(E′)** CN model shows the region (left of clipping plane) and stack direction (blue arrow) of panel **(E)**. **(F)** CP labeling with IgG outlines stroma rather than vessel lumen, reaching up to epithelial tight junctions, giving the CP a “spiny” appearance in single optical sections. **(F′)** Z-projection of CP, pseudocolored as panel **(A)**. 3D models are from mouse brain, and for qualitative reference only, since rat structures are similar but not identical.

After staining and clearing, brains were attached to a custom 3D-printed holder, then submerged in a 40 × 40 × 40 mm quartz cuvette (Portmann Instruments) filled with dibenzyl ether (DBE, nd = 1.562) and imaged using a home-built mesoscale single-plane illumination microscope (mesoSPIM). The microscope consists of a dual-sided excitation path using a fiber-coupled multiline laser combiner (405, 488, 515, 561, 594, 647 nm, Omicron SOLE-6) and a detection path comprising an Olympus MVX-10 zoom macroscope with a 1× objective (Olympus MVPLAPO 1×), a filter wheel (Ludl 96A350), and a scientific CMOS (sCMOS) camera (Hamamatsu Orca Flash 4.0 V3). The excitation paths also contain galvo scanners (GCM-2280-1500, Citizen Chiba) for light-sheet generation and reduction of shadow artifacts due to absorption of the light-sheet. In addition, the beam waist is scanned using electrically tunable lenses (ETL, Optotune EL-16-40-5D-TC-L) synchronized with the rolling shutter of the sCMOS camera. This axially scanned light-sheet mode (ASLM) leads to an uniform axial resolution across the field-of-view (FOV) of 4–10 μm (depending on zoom and wavelength). Image acquisition is done using custom software written in Python. Field of views ranged from 6.54 mm at 2× (Pixel size: 3.3 μm; as in Figures 1D, 2A,B), 3.27 mm at 4× (Pixel size: 1.6 μm; as in Figures 1A,C, 2C) to 2.62 mm at 5× (Pixel size: 1.28 μm; as in Figures 2E,F,F′). Z-stacks were acquired at 1 μm spacing (Figures 1A,C, 2E,F,F′) or 3 μm (all other panels). The laser/filter combinations were: Autofluorescence: 488 nm excitation and a 520/35 bandpass filter (BrightLine HC, AHF); Alexa 555: 561 nm excitation and 561 nm longpass (561LP Edge Basic, AHF); Alexa 633: 647 nm excitation and multiband emission filter (QuadLine Rejectionband ZET405/488/561/640, AHF). Further technical details of the custom SPIM will be described elsewhere^1^.

### Image Analysis

Autofluorescence subtraction, z-stacking and pseudocoloring was performed in FIJI (Schindelin et al., 2012); 3D segmentation was performed with the Threshold-based snake evolution algorithm of ITK-SNAP (Yushkevich et al., 2006). 3D models were oriented using Paraview (Ahrens et al., 2005) and the Allen Institute Brain Explorer.

## Results

After decalcification and clearing, the temporal bone became transparent enough to allow visualization of inner ear structures (Figure 1). The autofluorescence allowed identification of nonlabeled structures, such as the Organ of Corti (OC), stria vascularis (SV) and spiral ganglion (SG) neurons (Figures 1A,C), and was used for macroscopic structure segmentation (Figure 1B). In particular, we were able to follow and reconstruct the osseous channels connecting inner ear and CSF space, i.e., the endolymphatic duct (ED; Figures 1B,D) and cochlear aqueduct (CA; Figure 1B). Several macrophage populations were evident in the SG, nerve-associated blood vessels (Figure 1A), SV, spiral ligament (SL; Figure 1C), petrosal bone, and ED (Figure 1D).

In the brainstem, the meningeal fold surrounding the foramen of Luschka was clearly delineated (Figure 2A), and marked the passage from the 4th ventricle to subarachnoid space. Similarly, the dural fold covering the ED and sac was visible in association to the temporal bone (Figures 1D, 2B). Microglia and macrophages (Figures 2A–C) were clearly labeled throughout the sample. IgG labeling allowed vessel reconstruction throughout the whole DCN (Figures 2E,E′), and in all brain parenchyma. In the CP, however, the stroma was labeled (Figures 2F,F′), consistent with the presence of fenestrated endothelium permeable to macromolecules (Strazielle and Ghersi-Egea, 2013).

The CP was attached to the dorsal side of the 4th ventricle (Figure 2A) and filled the lateral ventricular expansion around the DCN (Figure 2B) in all rats observed (*n* = 6). Occasionally, a branch from the CP formed a “foot” structure, connected to the CP body through a thin stalk, that reached DCN surface and appeared to touch it (Figure 2C). Points of contact could be counted by automated segmentation of the ventricle volume surrounding DCN, where they appeared as holes (Figure 2D). The number of contacts was quite variable (from 0 to 13, *n* = 4), and the distribution was not tonotopic. Macrophages were observed in CP “feet”; colocalization with TTR, which labels CP epithelium, showed their localization in the stroma rather than on the CSF side (Figure 2C).

## Discussion

By combining decalcification and iDISCO+ clearing, we have obtained a rat preparation that allows visualization of cell populations in the auditory system from the cochlea to the brainstem while keeping all surrounding immune structures *in situ*. After observation of the macrophage populations in the auditory brainstem and periphery, three points are especially worth focusing on, all involving the microanatomy of neuroimmune gates.

First, the CP forms (seemingly random) close contacts with the DCN, where the plexus epithelium and the DCN surface appear to touch. This association is likely conserved from rat to human, since sporadic CP-DCN associations have been observed in autoptic human samples (Terr and Edgerton, 1985), and elucidating interactions in rodents may therefore yield new strategies for treating human hearing disorders. This is particularly interesting given that in the rat, upon cochlear ablation, the DCN displays an acute neuroinflammatory reaction mainly in its surface facing the ventricle (Perin et al., 2017), rather than in its deep layer receiving cochlear nerve fibers (Trussell and Oertel, 2017), and that CP trafficking of macrophages is observed after stroke (Ge et al., 2017) and spinal cord injury (Shechter et al., 2013).

Second, the CP also reaches the dural fold covering the ED and sac (which drains the inner ear endolymph into a dural sinus). Given that the dura is permeable to several inflammatory factors (Zhao et al., 2017), the observed configuration could play a role in the still largely unexplored relations between inner ear fluids and CP (Salt and Hirose, 2018). Perilymphatic spaces of the ear, on the other hand, are continuous with CSF spaces (Salt and Hirose, 2018), but so far their relations to meninges and CP have not been studied.

Finally, the cerebellopontine angle represents a unique brain region where CP and meninges are in close contact: all other ventricles are closed within the brain parenchyma, and other CPs only contact the ventricular surface. Given that the immune populations of meninges and CP differ in both roles and ontogeny (Prinz et al., 2017), and that CSF is regionally heterogeneous and involved in modulating neural responses (Veening and Barendregt, 2010) it appears highly likely that contacts between these two structures are regulated. The cleared preparation we have developed allows visualization of cell populations in this special neuroimmune assembly in several pathophysiological conditions.

## Data Availability

The datasets generated for this study are available on request to the corresponding author.

## Author Contributions

PP and RP contributed the biological idea and analyzed the images. PP performed the experiments and wrote the article. FV, PB and FH performed the lightsheet scans.

## Conflict of Interest Statement

The authors declare that the research was conducted in the absence of any commercial or financial relationships that could be construed as a potential conflict of interest.

## Footnotes

^1^www.mesospim.org

## Supplementary Material

FIGURE S1 Images of rat hindbrain clearing with iDISCO+. **(A)** Sample in PBS before clearing. Sagittal section is visible. BS, brainstem; CB, cerebellum; TB, temporal bone. **(B)** Cleared sample submerged in dibenzyl ether (DBE).
