# Supplementary figures and images for: iDISCO+ for the Study of Neuroimmune Architecture of the Rat Auditory Brainstem
Source: Front Neuroanat. 2019 Feb 13;13:15. doi: 10.3389/fnana.2019.00015 (PMC6381022; doi:10.3389/fnana.2019.00015)

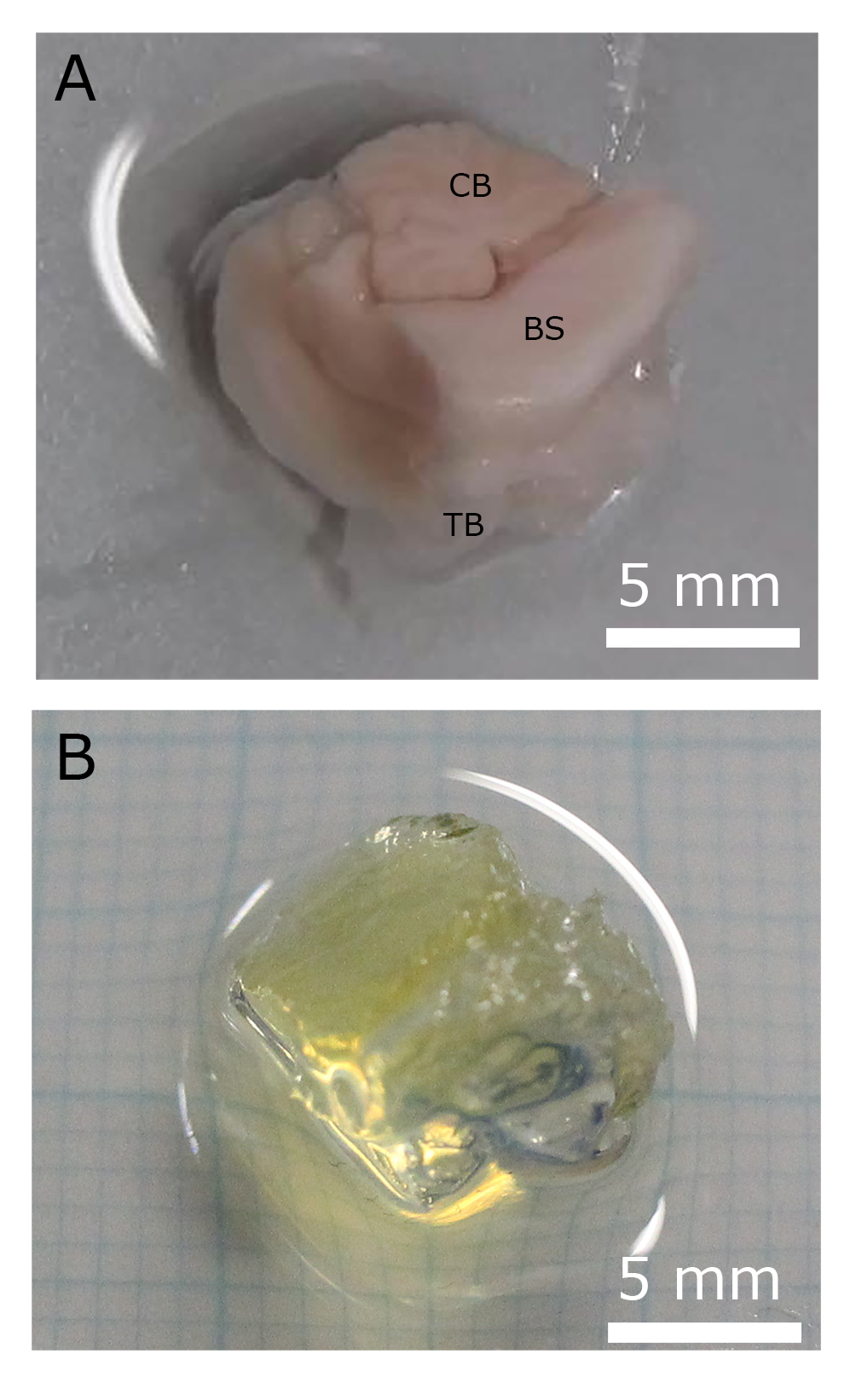

Supplement: FIGURE S1 — Images of rat hindbrain clearing with iDISCO+. (A) Sample in PBS before clearing. Sagittal section is visible. BS, brainstem; CB, cerebellum; TB, temporal bone. (B) Cleared sample submerged in dibenzyl ether (DBE). [file Image_1.TIF]
